# Do we need psychiatric asylums again? - Pro Speaker

**DOI:** 10.1192/j.eurpsy.2025.39

**Published:** 2025-08-26

**Authors:** M. Martin-Carrasco

**Affiliations:** Psychiatry, Clinica Psiquiatrica Padre Menni, Pamplona, Spain

## Abstract

**Abstract:**

Currently, there are few affordable, high-quality long-term care options available for a significant segment of people with serious mental disorders. This population includes adults characterized as poor insight, chronically ill, lacking autonomy, consuming drugs, and severe behavioural disturbances. These individuals frequently suffer from schizophrenia, bipolar disorder, toxic psychosis, severe personality disorders and substance abuse disorder, comorbidity being common. The void is ethically unacceptable and societally costly. Progressive reformers, consumers, civil libertarians, and health economists all advocated for a similar goal—the closure of publically funded psychiatric institutions. But deinstitutionalization has actually been trans-institutionalization. As psychiatric hospitals closed, patients with chronic psychiatric illnesses who lacked family support were moved into nursing homes or other forms of non-specialized residential care, occasionally visiting the psychiatric units of general hospitals. Many became homeless and intermittently used hospital emergency services for urgent care. Most disturbingly, prisons have become the largest mental health care facilities in some countries. The number of people affected may increase as a result of the crisis of the family institution, immigration and the epidemic of drug use.

Realistically, the deployment of both private and public resources is now imperative to provide appropriate care for a number of seriously mentally ill persons. They deserve a safe place to live with proper supports—not cycling between the streets, emergency departments, and prisons. Of course, psychiatric hospitals are a necessary but not sufficient component of a reformed spectrum of psychiatric services, where palliative psychiatry should have a role. A return to asylum based long-term psychiatric care will not remedy the complex problems of the mental health system, especially for patients with milder forms of mental illness who can thrive with high quality outpatient care. However, reforms that ignore the importance of expanding the role of such institutions will fail mental health patients who cannot safely live alone or care for themselves.

**Disclosure of Interest:**

None Declared

